# Imaging differentiation of solid pseudopapillary neoplasms and neuroendocrine neoplasms of the pancreas

**DOI:** 10.1016/j.ejro.2024.100576

**Published:** 2024-05-31

**Authors:** Ekaterina Khristenko, Matthias M. Gaida, Christine Tjaden, Verena Steinle, Martin Loos, Korbinian Krieger, Tim F. Weber, Hans-Ulrich Kauczor, Miriam Klauß, Philipp Mayer

**Affiliations:** a Clinic for Diagnostic and Interventional Radiology, Heidelberg University Hospital, Heidelberg 69120, Germany; b Institute of Pathology, University Medical Center Mainz, JGU-Mainz, Mainz 55131, Germany; c Joint Unit Immunopathology, Institute of Pathology, University Medical Center, JGU-Mainz and TRON, Translational Oncology at the University Medical Center, JGU-Mainz, Mainz 55131, Germany; d Institute of Pathology, Heidelberg University Hospital, Heidelberg 69120, Germany; e Department of General, Visceral, and Transplantation Surgery, Heidelberg University Hospital, Heidelberg 69120, Germany; f Department of Nuclear Medicine, Inselspital, University Hospital Bern, Bern 3010, Switzerland

**Keywords:** solid pseudopapillary neoplasm, Frantz tumor, pancreatic neuroendocrine neoplasm, pancreatic neuroendocrine carcinoma, computed tomography, magnetic resonance imaging

## Abstract

**Purpose:**

The present study aimed to compare the computed tomography (CT) and magnetic resonance imaging (MRI) features of solid pseudopapillary neoplasms (SPNs) and pancreatic neuroendocrine neoplasms (pNENs).

**Method:**

Lesion imaging features of 39 patients with SPNs and 127 patients with pNENs were retrospectively extracted from 104 CT and 91 MRI scans.

**Results:**

Compared to pNEN patients, SPN patients were significantly younger (mean age 51.8 yrs versus 32.7 yrs) and more often female (female: male ratio, 5.50:1 versus 1.19:1). Most SPNs and pNENs presented as well-defined lesions with an expansive growth pattern. SPNs more often appeared as round or ovoid lesions, compared to pNENs which showed a lobulated or irregular shape in more than half of cases (p<0.01). A surrounding capsule was detected in the majority of SPNs, but only in a minority of pNENs (<0.01). Hemorrhage occurred non-significantly more often in SPNs (p=0.09). Signal inhomogeneity in T1-fat-saturated (p<0.01) and T2-weighted imaging (p=0.046) as well as cystic degeneration (p<0.01) were more often observed in SPNs. Hyperenhancement in the arterial and portal-venous phase was more common in pNENs (p<0.01). Enlargement of locoregional lymph nodes (p<0.01) and liver metastases (p=0.03) were observed in some pNEN patients, but not in SPN patients. Multivariate logistic regression identified the presence of a capsule (p<0.01), absence of arterial hyperenhancement (p<0.01), and low patient age (p<0.01), as independent predictors for SPN.

**Conclusions:**

The present study provides three key features for differentiating SPNs from pNENs extracted from a large patient cohort: presence of a capsule, absence of arterial hyperenhancement, and low patient age.

## Highlights

• Solid pseudopapillary neoplasms (SPNs) are often larger and rounder than pancreatic neuroendocrine neoplasms (pNENs).
• The presence of a fibrous capsule is a strong imaging predictor of SPNs compared to pNENs.
• Arterial hyperenhancement is rare in SPNs, but present in over half of pNENs.
• Upstream dilatation of the pancreatic or bile duct almost never occurs in SPNs, but can occur in pNENs.

## Graphical Abstract

**Figure:**
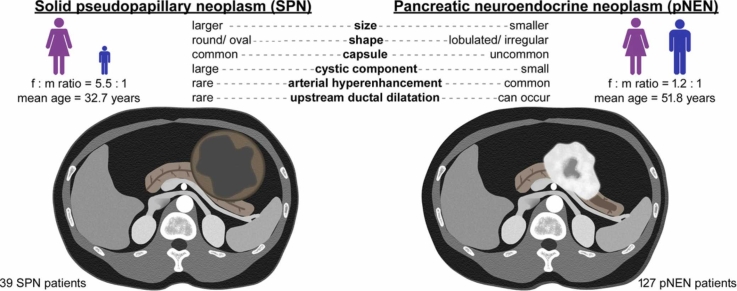

## 1 Introduction

Solid pseudopapillary neoplasms (SPNs; Frantz tumors) of the pancreas are uncommon pancreatic tumors with low malignant potential that have attracted increasing attention in recent years [1]. They were first well described by Virginia Frantz in 1959 [2] and account for 2–3 % of pancreatic neoplasms [3]. The tumorigenesis of SPN is controversial. Some studies conclude that SPNs could originate from pluripotent embryonic stem cells [4] while others favor an origin from neuroendocrine cells or from genital ridge-related cells which were incorporated into the pancreas during the process of organogenesis [5]. The correct preoperative diagnosis of SPNs can be challenging since SPNs can resemble features of other pancreatic tumors both histopathologically and radiologically [6]. The monomorphic tumor cells of SPNs look similar to endocrine cells [5] and often are positive for neuroendocrine markers like neuron-specific enolase and synaptophysin [6]. In imaging, both SPNs and pancreatic neuroendocrine neoplasms (pNENs) frequently present as well-circumscribed lesions that displace rather than invade adjacent structures, as opposed to pancreatic ductal adenocarcinomas (PDACs) that exhibit an infiltrative growth pattern [7], [8]. Similarly to large SPNs, large pNENs tend to undergo some degree of cystic and/ or calcific degeneration [9]. In clinical practice, strong contrast enhancement, most pronounced in the arterial phase, is often seen as the hallmark of pNENs [7]. However, previous studies reported that hyperenhancement can also be observed in SPNs [6], [10], [11] and can be absent in half of pNENs [12]. Although SPNs tend to occur in young women [1], [13] and pNENs are most often detected in middle-aged patients without clear gender predilection [7], there is a wide overlap in epidemiological variables [7], [14]. The clinical presentation of pNEN and SPNs is often similar as symptoms of hormone overproduction might be present only in a minority of pNENs [15]. Therefore, radiology plays a crucial role in distinguishing these two tumor entities, but radiological misdiagnosis is rather frequent [6], [10], [16], [17], [18], [19] and systematic radiological comparison studies are rare [20], [21], [22], [23].

The present study aimed to compare the computed tomography (CT) and magnetic resonance imaging (MRI) features of SPNs and pNENs.

## 2 Materials and methods

### 2.1 Patient population

The present retrospective single-center study was approved by the institutional review board of our institution (S-533/2018 [Oct 9th, 2018] and S-142/2023 [Mar 23rd, 2023]). The requirement of informed consent was waived. All procedures were performed in compliance with relevant laws and institutional guidelines. The work described has been carried out in accordance with The Code of Ethics of the World Medical Association (Declaration of Helsinki). The radiological information system (RIS) of our local university hospital was searched retrospectively for patients who had undergone a CT or MRI scan of the pancreas prior to resection of an SPN or pNEN between January 2005 and December 2019. Exclusion criteria were poor image quality (definitely noisy images, low spatial resolution) or strong artifacts (degrading diagnostic capability) [24] of the CT or MRI scan from a subjective assessment by a board-certified radiologist (not identical to the readers) and the time interval between CT or MRI scan and surgery of more than 6 months.

### 2.2 Image acquisition

Imaging protocols varied (see Supplementary Materials).

MR imaging was performed on scanners from Siemens Healthcare (Forchheim, Germany), Philips Healthcare (Best, Netherlands), GE Healthcare (New York, USA), and Canon Medical Systems (Ōtawara, Japan), with field strengths ranging from 1 to 3 Tesla.

The following imaging sequences were used for MR image analysis: Unenhanced T1- and T2-weighted images with and without fat saturation (fs), diffusion-weighted imaging (DWI) with b-value ≥ 500 s/mm^2^, and contrast-enhanced T1-weighted images (arterial phase, defined as full enhancement of hepatic arteries and absence of antegrade enhancement of hepatic veins, portal-venous phase, defined as full enhancement of portal veins and antegrade enhancement of hepatic veins, and late phase, defined as similar enhancement of portal veins and hepatic veins and enhancement of liver parenchyma to lesser degree than in portal venous phase). Available sequences with adequate image quality in SPN and pNEN patients are summarized in Supplementary Table 1.

CT imaging was performed on scanners from Siemens Healthcare, Philips Healthcare, GE Healthcare, and Canon Medical Systems, with 16–256 rows. The following imaging series were used for CT image analysis: Unenhanced phase (precontrast), arterial phase, portal-venous phase, and delayed phase. Kernels for image reconstruction were soft. Available CT series with adequate image quality in SPN and pNEN patients are summarized in Supplementary Table 2.

### 2.3 Imaging analysis

CT and MR images were independently analysed by two board-certified radiologists with more than 7 years of experience in abdominal imaging, each, blinded to the histopathological diagnoses, using the Digital Imaging and Communications in Medicine (DICOM) viewer mint Lesion (Mint Medical GmbH, version 3.7.3, Heidelberg, Germany) on a Picture Archiving and Communication System (PACS) workstation. Afterward, discrepancies in image interpretation were resolved by consensus between the two radiologists.

Imaging analysis included the following parameters: location (head, body, tail); size (in mm); shape (round or ovoid, lobulated or irregular); margin (sharp, irregular); presence of a capsule (present, absent; defined as peripheral rim with enhancement pronounced in the portal-venous and late phases on CT/MRI and with T2-hypointensity (without fs) on MRI [8], [22]); volume of any cystic component in relation to the volume of the complete lesion (≤ 25 %, >25 % and ≤75 %, >75 %); fluid-fluid-levels (present, absent); signal intensity/ CT density on the arterial, portal venous, and delayed phase relative to the surrounding pancreatic parenchyma (hypointense/ hypodense, isointense/ isodense, hyperintense/ hyperdense); encasement of any surrounding vessels ≥ 180° (present, absent); occlusion of any adjacent vessels (fresh thrombus, tumor thrombus, occlusion without thrombus, absent); definite evidence for invasion of adjacent organs (present, absent); dilatation of the main pancreatic duct (MPD) ≥ 4 mm and common bile duct (CBD) ≥ 8 mm upstream of the lesion were recorded (present, absent; for lesions without proximity to the MPD or CBD and for lesions located at the tip of the pancreatic tail, the latter two parameters were classified as “not applicable”); atrophy of upstream pancreatic parenchyma (present, absent; for lesions without proximity to the MPD and for lesions located at the tip of the pancreatic tail, this parameter was classified as “not applicable”).

The following parameters were assessed solely for MR images: Signal intensity (hypointense, isointense, hyperintense) and signal uniformity (homogeneous, heterogeneous) of the lesion on unenhanced T1- and T2-weighted imaging with and without fat saturation (fs) compared to the surrounding pancreas; the presence of T1-hyperintense spots with corresponding T2-hypointensity as evidence for hemorrhage (present, absent); signal intensity on DWI with b ≥ 500 s/mm^2^ compared to the surrounding pancreas (hypointense, isointense, hyperintense), apparent diffusion coefficient (ADC) values (avoiding cystic lesion parts and calcifications).

The following parameters were assessed solely for CT images: Calcification (peripheral, central, both, absent) and CT density on unenhanced phase (hypodense, isodense, hyperdense).

### 2.4 Statistical analysis

Statistical analysis was performed using MedCalc V22.016 (MedCalc Software, Ostend, Belgium). Agreement between radiologists was quantitated using Cohen’s kappa for nominal categorical variables [25] and weighted kappa (linear weights) for ordinal categorical variables [26]. As proposed by Landis and Koch [27], kappa values were interpreted as poor (< 0.00), slight (0.00 – 0.20), fair (0.21 – 0.40), moderate (0.41 – 0.60), substantial (0.61 – 0.80), and almost perfect agreement (0.81 – 1.00). Consistency of tumor size and ADC values between readers was quantitated using the intraclass correlation coefficient (ICC). Mann–Whitney U test was used for comparison of continuous variables between groups. For categorical variables, a chi-square test was used if no more than 20 % of the cells had expected frequencies < 5 and no cell had an expected frequency < 1 [28]. In other cases, the Fisher exact test was used for 2×2 frequency tables and the Freeman-Halton extension of the Fisher exact test was used for 2×3 frequency tables [29]. Multivariate analysis using logistic regression was performed for the discrimination between SPN and pNEN. Receiver operating characteristic (ROC) curves were employed to analyze the diagnostic performance of the contiguous parameters of patient age, tumor size, and the predicted probabilities of the logistic regression model in distinguishing SPN from pNEN. ROC curves were not computed for categorical variables [30]. The areas under the ROC curves (AUCs) with 95 % confidence intervals (CIs) were determined. All analyses were two-sided and p values < 0.05 were considered statistically significant.

## 3 Results

### 3.1 Patient characteristics

39 patients with SPNs (33 females and 6 males; mean age 32.7; range 13–70 years) were included in the study. Both preoperative CT and MRI scans were available in 2 SPN patients, solely preoperative MR images in 24 patients, and solely preoperative CT images in 13 patients.

127 patients with pNENs (69 females, 58 males; mean age 51.8 years, range 18–79 years) were included in the study. pNEN-lesions were pathologically graded as neuroendocrine tumor (NET) G1 in 60 cases, as NET G2 in 55 cases, as NET G3 in 7 cases, and as neuroendocrine carcinoma (NEC) G3 in 5 cases. 29 of the 127 pNEN (22.8 %) showed clinical symptoms related to hormone hypersecretion and were classified as having functioning pNEN. For 27 patients with pNEN, both preoperative CT and MRI scans were available. 38 pNEN patients solely had preoperative MRI and solely CT images were available in 62 pNEN patients.

**Fig. 1:**
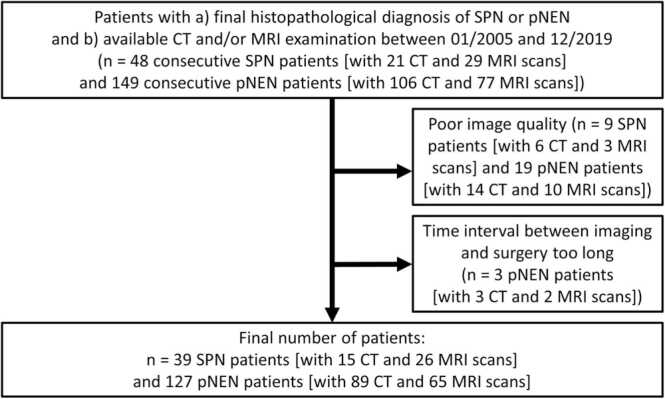
Flowchart of the study population.

A flowchart of the study population is presented in Fig. 1.

Age and sex distribution are shown in Supplementary Figure 1.

The proportion of female patients was significantly higher in the SPN group (33 out of 39 patients, 84.6 %) than in the pNEN-group (69 out of 127 patients, 54.3 %; p < 0.01).

SPN patients were significantly younger (mean age 32.7 years, range 13−70 years) than pNEN patients (mean age 51.8 years, range 18−79 years, p < 0.01).

Histopathological diagnosis was available for all patients.

### 3.2 Imaging interpretation

Median agreement between reader 1 and reader 2 ranged from substantial to almost perfect for ordinal categorical variables (median weighted kappa = 0.85, range 0.70–1.00) and from moderate to almost perfect for nominal categorical variables (median Cohen’s kappa = 0.87, range 0.57−1.00) (Table 1, Table 2). The ICC_ADC_ was 0.81. All discrepancies in image interpretation were resolved by consensus between the two readers. This was necessary for 3.5 % of individual values analyzed.

**Table 1:** Lesion imaging features.

**General imaging features**		**SPN**				**pNEN**				**p**	**k**
MR+CT	Location	head: 38.5 %%	body: 23.1 %		tail: 38.5 %	head: 29.1 %	body: 26.8 %		tail: 44.1 %	0.55	0.86
MR+CT	Median size (range)	4.2 cm (1.0 cm to 14.0 cm)				2.9 cm (0.7 cm to 15.7 cm)				**<0.01**	†
MR+CT	Shape	round / ovoid: 76.9 %		lobulated/ irreg.: 23.1 %		round/ ovoid: 43.3 %		lobulated/ irreg.: 56.7 %		**<0.01**	0.84
MR+CT	Volume of cystic component	≤25 %: 38.4 %	25–75 %: 35.9 %		>75 %: 25.6 %	≤25 %: 70.9 %	25–75 %: 15.0 %		>75 %: 14.2 %	**<0.01**	0.84
**Margin**		**SPN**				**pNEN**				p	**k**
		**distinct**		**indistinct**		**distinct**		**indistinct**			
MR	Margin	92.3 %		7.7 %		84.6 %		15.4 %		0.50	0.57
CT	Margin	86.7 %		13.3 %		73.0 %		27.0 %		0.26	0.81
**Capsule**		**SPN**				**pNEN**				**p**	**k**
		**none**	**<3 mm**		**≥3 mm**	**none**	**<3 mm**		**≥3 mm**		
MR	Capsule	30.8 %	34.6 %		34.6 %	89.2 %	9.2 %		1.5 %	**<0.01**	0.89
CT	Capsule	40.0 %	53.3 %		6.7 %	93.3 %	6.7 %		0.0 %	**<0.01**	0.93
**Relative signal intensity/density**		**SPN**				**pNEN**				**p**	**k**
		**hypo**	**iso**		**hyper**	**hypo**	**iso**		**hyper**		
MR	T1	95.5 %	0.0 %		4.5 %	95.1 %	3.3 %		1.6 %	0.72	0.79
MR	T1fs	100 %	0.0 %		0.0 %	94.4 %	3.7 %		1.9 %	0.99	0.79
MR	T2	0.0 %	4.0 %		96.0 %	1.6 %	42.2 %		56.2 %	**<0.01**	0.76
MR	T2fs	0.0 %	0.0 %		100 %	2.3 %	15.9 %		81.8 %	0.37	0.70
CT	Unenhanced	57.1 %	28.6 %		14.3 %	10.0 %	90.0 %		0.0 %	**<0.01**	0.79
MR	Arterial phase	100 %	0.0 %		0.0 %	23.2 %	26.8 %		50.0 %	**<0.01**	0.91
CT	Arterial phase	91.7 %	0.0 %		8.3 %	17.4 %	17.4 %		65.1 %	**<0.01**	0.97
MR	Portal venous phase	31.8 %	59.1 %		9.1 %	15.9 %	38.1 %		46.0 %	**<0.01**	0.90
CT	Portal venous phase	40.0 %	46.7 %		13.3 %	10.2 %	35.2 %		54.5 %	**<0.01**	0.95
MR	Delayed phase	30.0 %	50.0 %		20.0 %	15.8 %	50.9 %		33.3 %	0.30	0.78
CT	Delayed phase	0.0 %	100 %		0.0 %	0.0 %	50.0 %		50.0 %	0.23	0.75
MR	DWI (b ≥ 500 s/mm^2^)	0.0 %	0.0 %		100 %	0.0 %	8.0 %		92.0 %	0.99	1
**Homo-/heterogeneity**		**SPN**				**pNEN**				**p**	**k**
		**homo**		**hetero**		**homo**		**hetero**			
MR	T1	59.1 %		40.9 %		73.8 %		26.2 %		0.20	0.75
MR	T1fs	27.8 %		72.2 %		66.7 %		33.3 %		**<0.01**	0.86
MR	T2	16.0 %		84.0 %		38.1 %		61.9 %		**0.046**	0.79
MR	T2fs	14.3 %		85.7 %		38.6 %		61.4 %		0.11	0.88
**Other imaging features**		**SPN**				**pNEN**				**p**	**k**
		**present**		**absent**		**present**		**absent**			
MR+CT	Fluid-fluid-levels	2.6 %		97.4 %		0.8 %		99.2 %		0.42	0.83
MR	T1-hyperintense spots	16.0 %		84.0 %		4.7 %		95.3 %		0.09	0.92
CT	Calcification	cent.: 20.0 % peri.: 6.7 % both: 0.0 %		73.3 %		cent.: 7.9 % peri.: 1.1 % both: 5.6 %		85.4 %		0.14	0.93

Imaging features are stated for MR imaging and CT imaging. Imaging features without discrepancies between CT and MR imaging were pooled (MR+CT). Presented p values are from univariate analysis. Kappa values are for Cohen’s kappa or weighted kappa. † Intraclass correlation coefficient for lesion size = 0.95. Abbreviations specific to this table: cent.: central, hetero: heterogenous, homo: homogeneous, hyper: hyperintense/-dense, hypo: hypointense/-dense, irreg.: irregular, iso: isointense/-dense, peri.: peripheral.

**Table 2:** Associated imaging features.

**Modality**	**Feature**	**SPN**		**pNEN**		**p**	**k**
		**present**	**absent**	**present**	**absent**		
MR+CT	Vessel encasement	2.6 %	97.4 %	23.6 %	76.4 %	**0.01**	0.83
MR+CT	Vessel occlusion	FT: 0.0 % TT: 0.0 % w/oT: 7.7 %	92.3 %	FT: 0.0 % TT: 6.3 % w/oT: 14.2 %	79.5 %	0.14	0.97
MR+CT	Organ invasion	0.0 %	100 %	4.7 %	95.3 %	0.34	0.81
MR+CT	Upstream dilatation of common bile duct	0.0 %	100 %	22.0 %	78.0 %	**0.046**	0.93
MR+CT	Upstream dilatation of main pancreatic duct	3.6 %	96.4 %	29.7 %	70.3 %	**<0.01**	0.91
MR+CT	Atrophy of upstream parenchyma	0.0 %	100 %	30.7 %	69.3 %	**<0.01**	0.87
MR+CT	Multiple lesions	0.0 %	100 %	7.9 %	92.1 %	0.12	1
MR+CT	Lymph node enlargement	0.0 %	100 %	18.9 %	81.1 %	**<0.01**	0.91
MR+CT	Liver metastases	0.0 %	100 %	single: 2.4 % mult.: 12.6 %	85.0 %	**0.03**	1

All associated imaging features were not discrepant between MR and CT imaging. Therefore, associated imaging features were pooled for MR and CT imaging. Presented p values are from univariate analysis. Abbreviations specific to this table: FT: fresh thrombus, mult.: multiple, TT: tumor thrombus, w/oT: without thrombus.

In the following, the consensus values of the imaging features/ parameters for both readers are presented if not stated otherwise.

### 3.3 Imaging features

**Fig. 2:**
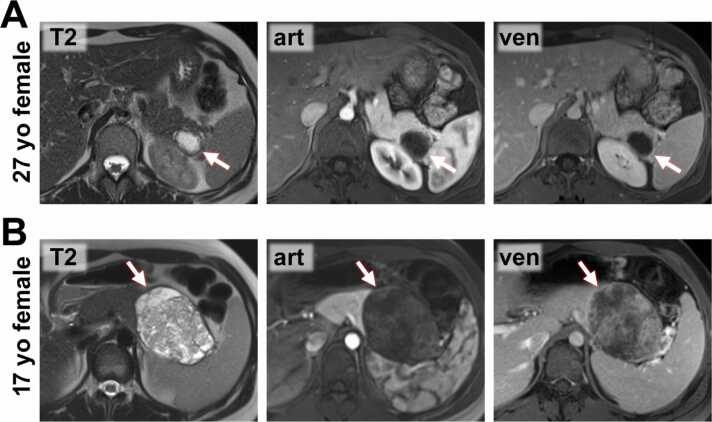
**MR Images from SPN patients. A)** 27-year-old female patient with an SPN in the tip of the pancreatic tail (*arrow*). The lesion is predominantly cystic with a continuous T2-hypointense capsule which shows enhancement most pronouncedly in the venous (ven) phase. **B)** 17-year-old female patient with a large SPN in the pancreatic tail (*arrow*). The lesion shows marked cystic degeneration and is surrounded by a continuous T2-hypointense capsule. The solid components of the lesion enhanced most pronouncedly in the venous phase.

**Fig. 3:**
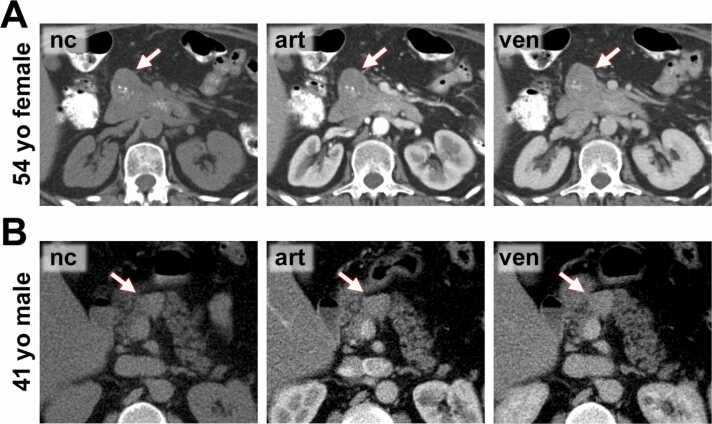
**CT images from SPN patients. C)** 54-year-old female patients with an SPN in the pancreatic head (*arrow*). The lesion is predominantly cystic with central calcifications (best seen in the non-contrast (nc) phase) and shows a capsule-like enhancement in the venous phase. **D)** 41-year-old male patient with a small SPN in the pancreatic body (*arrow*). The lesion is lesion is moderately hyperdense in the non-contrast, arterial (art), and venous phase. No capsule is apparent in CT. This lesion is very difficult to distinguish from pNEN.

**Fig. 4:**
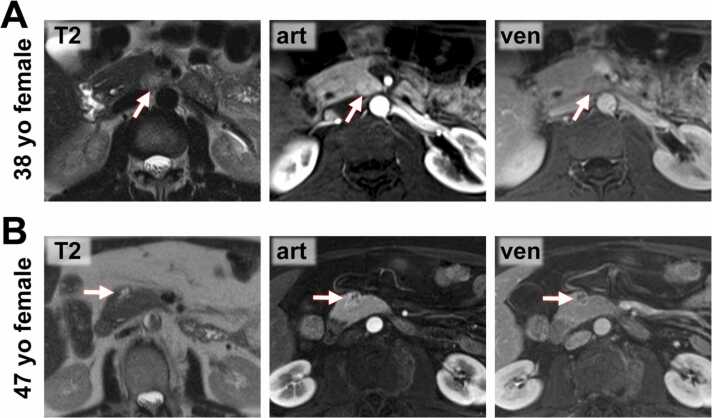
**MR images from pNEN patients. A)** 38-year-old female patient with a small pNEN in the medial part of the pancreatic head/ uncinated process (*arrow*). The lesion lacks a definite T2-hypointense capsule. It shows moderate arterial (art) hyperenhancement. **B)** 47-year-old female patient with a small pNEN in the pancreatic head with central cystic degeneration, lack of a definite T2-hypointense capsule, and marked enhancement in the arterial and venous (ven) phase (*arrow*).

**Fig. 5:**
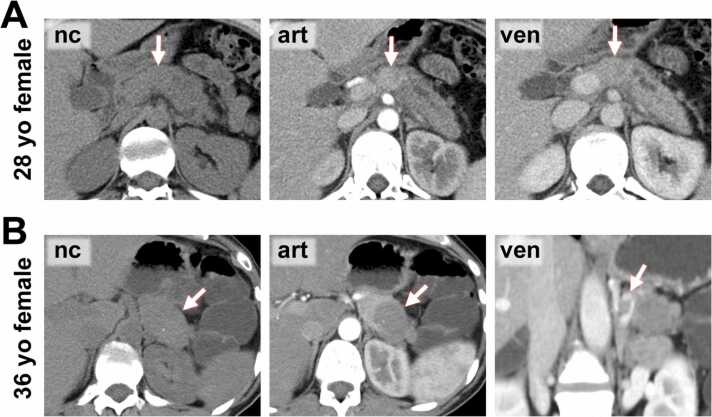
**CT images from pNEN patients. C)** 28-year-old female patient with a pNEN in the pancreatic body (*arrow*) which shows clear hyperdensity in the arterial phase and is associated with upstream dilatation of the main pancreatic duct. **D)** 36-year-old female patient with a pNEN with large cystic parts and a small calcification in the pancreatic tail (*arrow*). The encasement of the splenic vessels (depicted in the venous phase image) is helpful for differentiation from SPN.

Imaging features are summarized in Table 1, Table 2, and presented in Fig. 2, Fig. 3 for SPNs and Fig. 4, Fig. 5 for pNENs.

Most SPNs and pNENs appeared as well-defined lesions with an expansive growth pattern. Some pNENs showed signs of invasive growth with invasion of adjacent organs which was not observed in SPNs, although the difference was not significant (p = 0.34). pNENs were more frequently associated with upstream dilatation of the main pancreatic duct (p < 0.01) or obstructive cholestasis (p = 0.046). SPNs more often presented as round/ovoid lesions, compared to pNENs which showed a lobulated/irregular shape in more than half of cases (p < 0.01). pNENs as well as SPNs appeared as relatively hypointense lesions on unenhanced T1-weighted imaging and often showed hyperintensity on T2-weighted images, whereas the latter was more common in SPNs (p < 0.01). Cystic degeneration, although present in a relevant percentage of both tumor entities, was more often observed in SPNs than in pNENs (p < 0.01). SPNs were non-significantly more often associated with intratumoral hemorrhage (p = 0.09) and calcification (p = 0.14). Compared to PNENs, SPNs were more often inhomogeneous in T1fs- (p < 0.01) and T2-weighted imaging (p = 0.046), while differences were non-significant for T1- (p = 0.20) and T2fs-weighted imaging (p = 0.11). In unenhanced CT imaging, pNENs typically presented as isodense to the surrounding pancreatic parenchyma, whereas the majority of SPNs were hypodense in the unenhanced phase (p < 0.01). A surrounding capsule was detected in a relevant percentage of SPNs, but only in a minority of pNENs (p < 0.01). Hyperenhancement in the arterial phase was present in 50.0 % of pNENs in MR-imaging and 65.1 % of pNENs in CT-imaging. Only one SPN showed mild hyperdensity in arterial phase CT-imaging (p < 0.01). PNEN were significantly more often hyperintense/-dense in the portal-venous phase (p_MRI_ < 0.01, p_CT_ < 0.01) and non-significantly more often hyperintense/-dense in the delayed phase (p_MRI_ = 0.30, p_CT_ = 0.23). Available b-values varied (median lowest b-value 50 s/mm², range 0–50 s/mm²; median highest b-value 800 s/mm², range 600–1000 s/mm²). Hyperintensity in DWI with high b-values was common in both tumor entities. ADC values were similar for SPNs (median 1072 µm^2^/s, range 759–1416 µm^2^/s) and pNENs (median 1070 µm^2^/s, range 810–1307 µm^2^/s) (p = 0.80, non-significant). In two pNEN patients, only high b-value images were available and calculation of ADC values was not possible. Enlargement of locoregional lymph nodes (p < 0.01) and liver metastases (p = 0.03) were observed in some pNEN patients, but not in SPN patients.

Additional analyses can be found in the Supplementary Materials: the diagnostic performance of imaging and demographic features in distinguishing SPN from pNEN (Supplementary Table 3), the association of imaging features with histopathological grading in pNEN, and comparisons of imaging features of SPN and pNEN excluding G3 NET/NEC (Supplementary Tables 4 and 5).

**Fig. 6:**
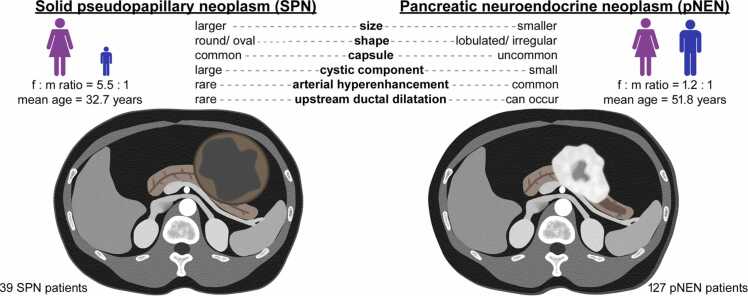
**Graphical summary.** Synopsis of the most relevant distinguishing imaging features.

The most relevant distinguishing imaging features are summarized in Fig. 6.

### 3.4 Discrepancies in imaging feature assessment for CT and MRI

In nine out of the twenty-seven pNEN patients for whom both CT and MRI scans were available, one or two imaging features per patient were discrepantly assessed for the two imaging modalities. In three pNEN patients, arterial hyperenhancement was detected by CT, but not MRI. In one patient, a pNEN showed arterial hyperenhancement in MRI, but not in CT. Three pNEN appeared predominantly hyperintense in the portal-venous phase in CT and hypo- or isointense in MRI. In two pNEN lesions, a capsule was visible solely in MRI, and in one pNEN lesion solely in CT. Four pNEN lesions which appeared to have indistinct margins in CT had clearly defined margins in MRI. One SPN lesion had indistinct margins in CT, but sharp margins in MRI.

### 3.5 Multivariate logistic regression model

Moreover, we employed a multivariate logistic regression model for imaging and demographic parameters discriminating SPN from pNEN. To make the model suitable for most patients, we selected independent variables which are applicable for both CT and MRI, which can be applied irrespective of the tumor location, and which have a prevalence of ≥ 20 % for at least one lesion entity. Independent variables were only selected if p < 0.05 from univariate analysis, except for tumor margin which had been found a good discriminating feature in a previous study [31]. The following independent variables were selected: patient age, sex, tumor size, tumor shape (round/ovoid versus lobulated/irregular), tumor margin (distinct versus indistinct), volume of cystic component (≤25 % versus >25 %), arterial enhancement (non-hyperdense/-intense vs. hyperdense/-intense), venous enhancement (non-hyperdense/-intense vs. hyperdense/-intense), capsule (present or absent), encasement of vessels (present or absent). 138 patients were included in the multiple regression analysis. Cases missing one of these variables, for instance cases without arterial phase imaging, as well as cases with discrepantly assessed imaging features for CT and MRI were not included in the multiple regression analysis. Using the forward method, variables were entered into the model if p < 0.05 and removed if p > 0.1. Low patient age, absence of arterial hyperenhancement, and presence of a capsule remained statistically significant predictors for SPN.

The results of the multivariate analysis using logistic regression are summarized in Table 3 and Supplementary Figure 2. The results from a multivariate analysis excluding G3 NET/NEC can be found in Supplementary Table 6.

**Table 3:** Multivariate analysis using logistic regression for features discriminating SPNs from pNENs.

	**Coefficient**	**Standard Error**	**P**	**Odds ratio (95 % CI)**
**Age [years]**	-0.0819	0.0251	<0.01	0.92 (0.88 – 0.97)
**Absence of arterial hyperenhancement**	2.950	1.077	<0.01	19.10 (2.31 – 157.81)
**Presence of a capsule**	2.484	0.691	<0.01	11.99 (3.10 – 46.41)
**Constant**	-0.910	1.423	0.52	

Overall model significance level p < 0.01. Proportion of cases correctly classified: 89.1 %. ROC curve analysis: AUC = 0.93 (95 % CI 0.88 – 0.97). Variables not included in the model: sex, tumor size, tumor shape, tumor margin, volume of cystic component, venous enhancement, encasement of vessels.

## 4 Discussion

In the present study, which is one of the largest comprehensive radiological studies on SPN and pNEN, we retrospectively analyzed 104 CT scans and 91 MRI scans from 39 patients with SPNs and 127 patients with pNENs. We identified three independent discriminating features for the differentiation of SPN from pNEN. First, the presence of a capsule was predictive of SPN. Second, arterial hyperenhancement was significantly less common in SPN. Third, SPN patients were significantly younger than pNEN patients.

These discriminating features can be easily applied to routine diagnostics. They can help to avoid the rather frequent imaging misdiagnosis of these relatively uncommon pancreatic tumor entities which show a significant overlap of epidemiological, clinical, and imaging features [1], [6], [10], [14], [15]. They both can appear as completely solid lesions, especially when they are small, while larger lesions tend to undergo some degree of cystic degeneration [5], [9].

One of the typical histopathological features of SPNs is a fibrous capsule which separates the tumor from the pancreatic parenchyma as well as other surrounding structures [3] and is reported to be present at histopathological examination in two-thirds to almost all SPN cases [32]. pNENs, however, although typically well-circumscribed, lack a defined capsule in the vast majority of cases [7]. Accordingly, a capsule was detected at cross-sectional imaging in well above half of our SPN cases and only in a minority of pNEN cases. Our data show that the presence of a capsule is a key imaging feature of SPNs which remained statistically significant in multivariate analysis. The imaging prevalence of a capsule was similar to those reported by others [8], [21], [33]. The superior soft tissue contrast of MRI is probably responsible for the slightly higher detection rate of a capsule by MRI than by CT [9]. One study reported at least partial encapsulation of pNENs in CT imaging in more than half of the cases [23] which is in contradiction to both the results from the present study and previous reports [7].

We show that the absence of arterial hyperenhancement is a strong predictor for SPN versus pNEN. Similarly as described by Karmazanovsky et al. [34], we observed arterial hyperenhancement in more than half of the pNEN lesions. This hallmark imaging feature of pNEN is attributed to their usually rich capillary bed and is closely correlated with intratumoral microvessel density [35]. By contrast, only one SPN lesion from our study exhibited mild hyperdensity in arterial phase CT imaging. However, several factors preclude the use of arterial enhancement as the sole criterion for the differentiation of SPN and pNEN. In some case series, the prevalence of arterial hyperenhancement in SPNs was more than 10 % [6] and up to half of pNENs may appear hypovascular at imaging [34]. Moreover, arterial hyperenhancement is an imaging feature with variable reproducibility. In the present study, hyperenhancement of a few pNEN-lesions was detected by CT, but not MRI, or vice versa. A probable explanation for this is that the tumor–parenchyma contrast depends markedly on the exact timing of the arterial and portal-venous phase [36]. In some hypervascular pNEN, the time window for the detection of hyperenhancement is less than ten seconds [36].

In our study, arterial hypoenhancement was more commonly observed in high-grade NET and NEC than in low-to-intermediate grade NET which is in line with a study by Kang et al. [37]. Importantly, arterial phase hypoenhancement and low microvessel density were also reported to be risk factors for the development of liver metastases and predictors of poor overall survival in pNEN [38], [39].

In the present study, cystic degeneration was more pronounced in the SPN group although the difference was only significant in univariate analysis. Opposed to the more frequent pancreatic ductal adenocarcinomas (PDACs), both SPNs and pNENs are reported to be well-circumscribed and displace rather than invade adjacent structures [7], [8]. In the present study, SPNs and pNENs tended to have distinct margins in CT as well as MR imaging whereas non-clearly defined margins were non-significantly more common in pNENs, especially high-grade (G3) NET and NEC.

Previous data from a small study by Zhu et al. indicated that SPNs typically appear as round or ovoid lesions whereas pNENs are more often lobulated or irregular in shape [23]. Our data confirm these previous results and indicate that a round or ovoid shape is a discriminator for distinguishing SPN from pNEN, although there is a broad overlap regarding this imaging feature. Remarkably, a lobulated or irregular shape was particularly typical for high-grade pNENs, as opposed to low-to-intermediate grade pNEN, and could reflect different growth rates of tumor cells in different parts of these more aggressive lesions.

SPNs typically affect young women while pNENs are most often detected in middle-aged patients without clear gender predilection. In our study, patient age was an independent discriminator between these two tumor entities even though the mean age of our SPN patients was higher than reported in a large meta-analysis (32.7 years versus 22.0 years) [14] and our pNEN patients were on average younger than in a large epidemiological study (51.8 years versus 58.5 years) [15]. As for the other independent discriminators, however, patient age should only be applied in conjunction with other features since we observed a marked overlap of age ranges (13–70 years for SPN, 18–79 years for pNEN).

Few studies have analyzed the potential of MRI texture features and radiomics to differentiate SPN and pNEN [40], [41]. In a study by Li et al., MRI texture analysis had good discrimination ability between SPN and nonfunctional pNEN [41]. Song et al. reported that their MRI-based radiomics approach showed sufficient performance for discriminating SPN and hypovascular nonfunctional pNEN [40]. Texture analysis and radiomics are powerful techniques that can go beyond human-eye-based semantic descriptors and allow the extraction of quantitative imaging features that can be used as discriminators between tumor entities. However, these techniques are not implemented into clinical practice yet [42].

There were limitations to our study. First, although the present study is larger than many other single center imaging studies on SPN and/or PNEN, the absolute sample size is still rather small. Due to the long recruitment period, there was considerable heterogeneity regarding imaging technique and quality as well as clinical management that changed over time, which could have induced substantial bias. The unavailablity of some imaging series in several patients could have led to missing imaging differences between patient groups, but well reflects clinical reality. Second, only surgically resected tumors were included. This may bias our study sample towards larger and higher-grade pNENs since some small low-grade pNENs are diagnosed by fine needle aspiration (FNA) and undergo surveillance. We did not include the latter lesions due to possible misdiagnosis of SPN as pNEN by FNA [43]. Third, our analysis of vessel encasement/ occlusion included any surrounding vessel and was not intended to define the surgical resectability status of the lesion, but rather to obtain information on lesion invasiveness.

## 5 Conclusions

In conclusion, the present study provides three key features for the differentiation of SPN and pNEN. First, pNENs, although commonly well-circumscribed, usually lack a definite capsule which, on the other hand, is a radiological key feature of SPNs. Second, arterial hyperenhancement, present in only a minority of SPNs, but more than half of pNENs, is a strong discriminating feature. Third, SPN patients are on average markedly younger than pNEN patients. Importantly, these key features should only be used collectively due to wide overlaps in imaging and clinical features between these two tumor entities.

## Ethical statement

The study was approved by the institutional review board of our institution (S-533/2018 [Oct 9th, 2018] and S-142/2023 [Mar 23rd, 2023]). The requirement of informed consent was waived. All procedures were performed in compliance with relevant laws and institutional guidelines. The work described has been carried out in accordance with The Code of Ethics of the World Medical Association (Declaration of Helsinki).

## CRediT authorship contribution statement

**Philipp Mayer:** Writing – original draft, Visualization, Project administration, Methodology, Investigation, Funding acquisition, Formal analysis, Conceptualization. **Christine Tjaden:** Writing – review & editing, Data curation. **Verena Steinle:** Writing – review & editing, Data curation. **Matthias M Gaida:** Writing – review & editing, Methodology, Data curation. **Ekaterina Khristenko:** Writing – review & editing, Investigation. **Hans-Ulrich Kauczor:** Writing – review & editing, Resources, Funding acquisition. **Tim F Weber:** Writing – review & editing, Conceptualization. **Korbinian Krieger:** Writing – review & editing, Data curation. **Martin Loos:** Writing – review & editing, Supervision. **Miriam Klauß:** Writing – review & editing, Supervision, Project administration.

## Declaration of Competing Interest

The authors declare that they have no known competing financial interests or personal relationships that could have appeared to influence the work reported in this paper.

## Data Availability

The datasets generated during and/or analyzed during the current study are available from the corresponding author upon reasonable request. The Digital Imaging and Communications in Medicine (DICOM) files cannot be made freely available due to privacy restrictions.
