# Supplementary material for: Imaging differentiation of solid pseudopapillary neoplasms and neuroendocrine neoplasms of the pancreas
Source: Eur J Radiol Open. 2024 May 31;12:100576. doi: 10.1016/j.ejro.2024.100576 (PMC11176946; doi:10.1016/j.ejro.2024.100576)
Supplement: Supplementary file 1 — Supplementary material [file mmc1.pdf]

Imaging differentiation of solid pseudopapillary neoplasms and neuroendocrine neoplasms of the pancreas

Supplementary Material

Imaging protocols

There were substantial variations in the imaging protocols due to the long study period (15 years) and due to the inclusion of external CT and MRI scans (our hospital is a tertiary referral center).

The most recent pancreatic CT protocol at our institution includes at least an arterial phase scan of the upper or whole abdomen and a portal venous phase of the whole abdomen, using a dual-source dual-energy CT scanner (SOMATOM Force, Siemens Healthineers, Forchheim, Germany). 80 ml of iodinated contrast agent (monophasic bolus, adjusted for small or large patients, 300mg/ml iodine concentration) followed by a 50 ml NaCl chaser bolus are injected via a cubital vein at 4ml/s. After a trigger threshold of 140 Hounsfield units in the abdominal aorta is reached, the arterial and portal venous CT acquisitions are initiated with a delay of 10-15 s (depending on the indication of the CT exam) and 45 s, respectively.

The most recent pancreatic MRI protocol includes at least non-contrast T1- and T2-weighted images with and without fat saturation (fs), chemical shift imaging, diffusion-weighted imaging (DWI) with b-values of 50, 400, and 900 s/mm^2, magnetic resonance cholangiopancreatography, and contrast-enhanced T1fs-weighted images, using a 3 Tesla scanner (MAGNETOM Vida, Siemens Healthineers). The contrast agent gadoterate meglumine (0.2ml/kg body weight) and a subsequent 30 ml NaCl chaser bolus are injected via a cubital vein at 2ml/s. The arterial acquisition is started using a bolus-tracker. The portal venous phase is acquired 70 s after contrast injection and the delayed phase usually 180 s after contrast injection.

Supplementary Table 1. Available MR imaging data

	Available in number of SPN patients	Available in number of pNEN patients
Unenhanced T1-weighted imaging without fs	22 (84.6 %)	61 (93.8 %)
Unenhanced T1-weighted imaging with fs	18 (69.2%)	54 (83.1 %)
Unenhanced T2-weighted imaging without fs	25 (96.2 %)	64 (98.5 %)
Unenhanced T2-weighted imaging with fs	14 (53.8 %)	44 (67.9%)
DWI with b ≥ 500 s/mm^2	9 (34.6 %)	25 (38.5 %)
Arterial phase imaging	14 (53.8 %)	56 (86.2 %)
Portal venous phase imaging	22 (84.6 %)	63 (96.9%)
Late phase imaging	20 (76.9 %)	57 (87.7 %)

In brackets are the percentages referring to all included MRI scans of the respective lesion entity.

Supplementary Table 2. Available CT imaging data

	Available in number of SPN patients	Available in number of pNEN patients
Unenhanced phase imaging	7 (46.7 %)	60 (67.4 %)
Arterial phase imaging	12 (80.0 %)	86 (96.6 %)
Portal venous phase imaging	15 (100 %)	88 (98.9 %)
Late phase imaging	3 (20.0 %)	12 (13.5%)

In brackets are the percentages referring to all included CT scans of the respective lesion entity.

Diagnostic performance of imaging and demographic features in distinguishing solid pseudopapillary neoplasms (SPNs) from pancreatic neuroendocrine neoplasms (pNENs)

According to Youden's J statistic, the presence of arterial hypoenhancement in MR had the highest performance (J = 0.77, 95% CI 0.40 – 0.87) in distinguishing SPNs from pNENs, followed by arterial hypoenhancement in CT (J = 0.74, 95% CI 0.34 – 0.90) (Supplementary Table 3). Arterial hypoenhancement had high negative predictive values (NPV, 100.0%, 95% CI 100.0 – 100.0 for MRI; 98.6%, 95% CI 91.6 – 99.8% for CT) and relatively low positive predictive values (PPV, 51.9%, 95% CI 40.1 – 63.4% for MRI; 42.3%, 95%CI 31.0 – 54.5% for CT). The presence of a capsule in MRI or CT had high negative NPVs (87.9%, 95%CI 80.2 – 92.8% for MRI; 93.3%, 95%CI 88.1 – 96.1% for CT) and moderate PPVs (72.0%, 95%CI 55.0 – 84.4% for MRI; 60.0%, 95%CI, 38.4 – 78.3%) as well as J values (0.58, 95%CI 0.27 – 0.81 for MRI; 0.53, 95%CI 0.18 – 0.81).

According to receiver operating characteristic (ROC) curve analyses, patient age had a high area under the curve (AUC) (0.85, 95%CI 0.78 – 0.90) while tumor size had a relatively low AUC (0.66, 95%CI 0.58 – 0.73) for differentiation of SPNs from pNENs. Both AUC values were significantly different from 0.5 ($p < 0.01$). According to Youden's J statistic, the optimal cut-off values were "≤ 41 years" for patient age (J = 0.59, 95% CI 0.35 to 0.76) and "> 21 mm" for tumor size (J = 0.28, 95% CI 0.05 – 0.46).

The sensitivity, specificity, positive predictive values (PPV), negative predictive values (NPV), and accuracy of selected radiological and demographical parameters are presented in Supplementary Table 3. ROC curves for patient age and tumor size are shown in Supplementary Figure 2.

Supplementary Table 3. Diagnostic performance of selected imaging and demographic features for differentiation of SPNs from pNENs (with 95% confidence intervals).

	Sensitivity [%]	Specificity [%]	PPV [%]	NPV [%]	Accuracy [%]
**Size**					
> 15 mm	94.9 (82.7 – 99.4)	25.2 (17.9 – 33.7)	28.0 (25.6 – 30.6)	94.1 (80.1 – 98.5)	41.6 (34.0 – 49.5)
> 21 mm †	87.2 (72.6 – 95.7)	40.9 (32.3 – 50.0)	31.2 (27.3 – 35.4)	91.2 (81.7 – 96.0)	51.8 (43.9 – 59.6)
> 29 mm	74.4 (57.9 – 87.0)	50.4 (41.4 – 59.4)	31.5 (26.3 – 37.3)	86.5 (78.5 – 91.8)	56.0 (48.1 to 63.7)
**Shape**					
Round/ oval	76.9 (60.7 – 88.9)	56.7 (47.6 – 65.5)	35.3 (29.5 – 41.5)	88.9 (81.6 – 93.5)	61.4 (53.6 – 68.9)
**Volume of cystic components**					
≥ 25 % †	61.5 (44.6 – 76.6)	70.9 (62.1 – 78.6)	39.3 (31.9 – 48.4)	85.7 (79.9 – 90.1)	68.7 (61.0 – 75.6)
> 75 %	25.6 (13.0 – 42.1)	85.8 (78.5 – 91.4)	35.7 (21.9 – 52.4)	79.0 (75.5 – 82.1)	71.7 (64.2 – 78.4)
**Capsule**					
Present in MRI †	69.2 (48.2 – 85.7)	89.2 (79.1 – 95.6)	72.0 (55.0 – 84.4)	87.9 (80.2 – 92.8)	83.5 (74.3 – 90.5)
Present in CT	60.0 (32.3 – 83.7)	93.3 (85.9 – 97.5)	60.0 (38.4 – 78.3)	93.3 (88.1 – 96.1)	88.5 (80.7 – 93.9)
**Arterial hypoenhancement**					
Present in MRI †	100.0 (76.8 – 100.0)	76.8 (63.6 – 87.0)	51.9 (40.1 – 63.4)	100.0 (100.0 – 100.0)	81.4 (70.3 to 89.7)
Present in CT	91.7 (61.5 – 99.8)	82.6 (72.9 – 89.9)	42.3 (31.0 – 54.5)	98.6 (91.6 – 99.8)	83.7 (74.8 – 90.4)
**Age**					
≤ 30 years	59.0 (42.1 – 74.4)	92.9 (87.0 – 96.7)	71.9 (56.4 – 83.5)	88.1 (83.5 – 91.5)	84.9 (78.6 – 90.0)
≤ 41 years †	79.5 (63.5 – 90.7)	79.5 (71.5 – 86.2)	54.4 (45.0 – 63.5)	92.7 (87.1 – 95.9)	79.5 (72.6 – 85.4)
≤ 54 years	97.4 (86.5 – 99.9)	44.9 (36.1 – 54.0)	35.2 (31.5 – 39.0)	98.3 (89.1 – 99.7)	57.3 (49.3 – 64.9)
**Sex**					
Female	84.6 (69.5 – 94.1)	45.7 (36.8 – 54.7)	32.4 (28.0 – 37.1)	90.6 (81.9 – 95.4)	54.8 (46.9 – 62.5)

For calculating positive predictive values (PPV), negative predictive values (NPV), and accuracy, the ratio of SPN and PNEN cases in the present cohort were presumed to reflect the prevalence of these diseases in the total population. Since predictive values and accuracy depend on the prevalence of the diseases, they are not transferable to populations with a different ratio of SPN and pNEN cases. A higher prevalence of SPNs would lead to an increase in PPV-values as well as a decrease in NPV-values and

vice versa, while the relationship between accuracy and prevalence is more complex [44]. *Cut-off values that yielded the highest Youden J statistics are marked with †.*

Supplementary Figure 1. Age distribution, sex distribution and ROC curves for age and tumor size.

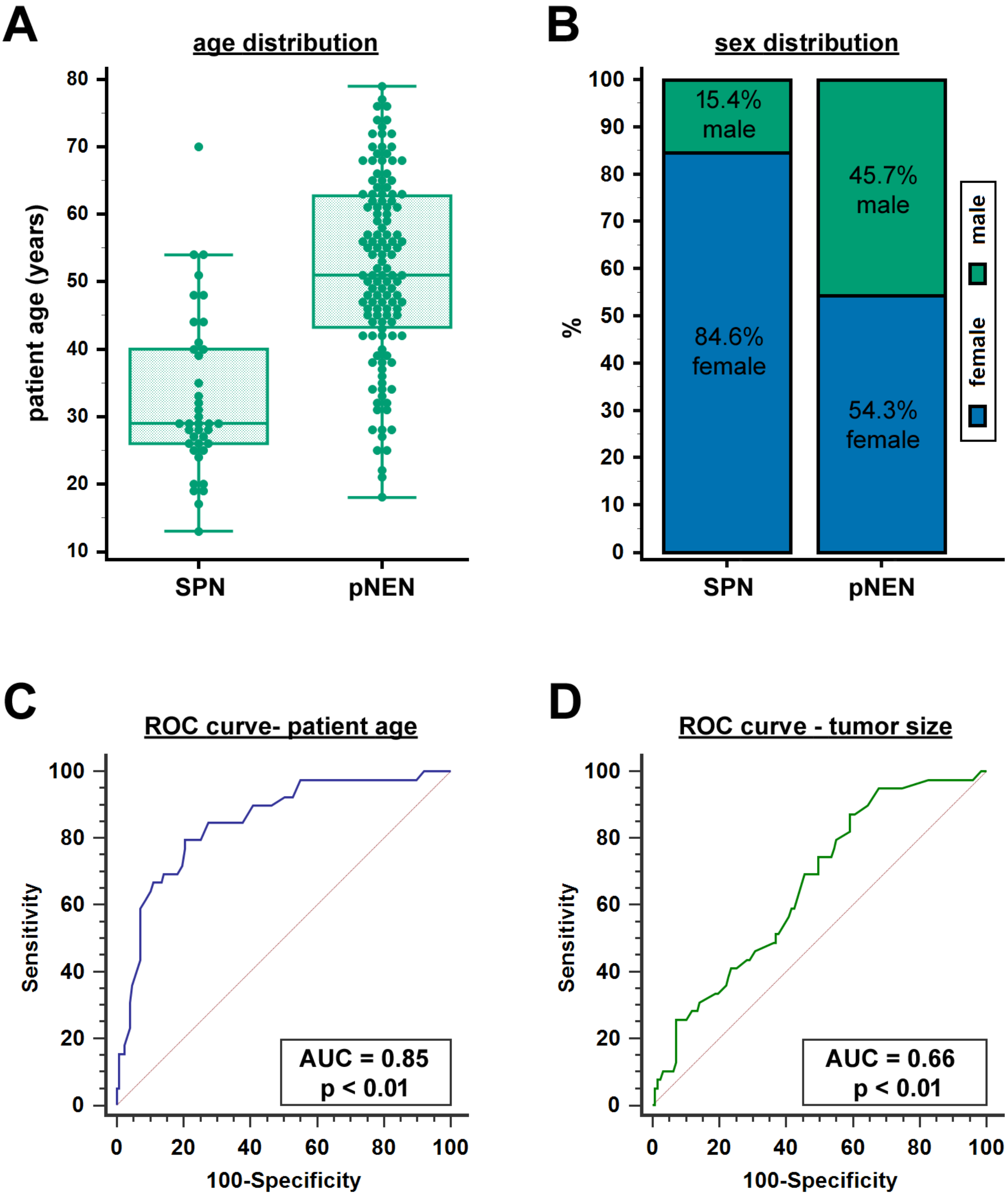

***A)** Box-and-whisker plot of the age distribution of SPN and pNEN patients. **B)** Stacked column chart of sex distribution of SPN and pNEN patients. **C)** ROC curve for differentiation of SPNs from pNENs using patient age. **D)** ROC curve for differentiation of SPNs from pNENs using tumor size.*

Association of imaging features with histopathological grading in pNENs

High-grade (G3) neuroendocrine tumors (NETs) and NECs were less likely to show hyperenhancement in the arterial and portal venous phase than low (G1) or moderate-grade (G2) NETs ($p < 0.01$). G3 NETs and NECs were more often ill-defined, but the difference was not statistically significant ($p_{MRI} = 0.11$, $p_{CT} = 0.24$). Encasement and occlusion of adjacent vessels ($p < 0.01$), upstream dilatation of the pancreatic main duct ($p < 0.01$) with concomitant parenchymal atrophy ($p < 0.01$), locoregional lymphadenopathy ($p = 0.01$) and hepatic metastases ($p < 0.01$) were also observed in a significantly higher percentage of G3 NETs and NECs than G1/G2 NETs. A lobulated or irregular shape was non-significantly more common in high-grade lesions (G3) than in low or moderate-grade lesions (G1/G2) ($p = 0.07$). Two out of two G3 NETs with available DWI were hyperintense in the high b-value images; in only one of these a low b-value (0 s/mm^2) was available and the mean apparent diffusion coefficient (ADC) value for both readers was 918 µm^2/s (⟷ median ADC for all pNENs = 1070 µm^2/s).

Supplementary Table 4. Lesion imaging features of SPN and pNEN (excluding G3 NET/NEC)

General imaging features		SPN			pNEN (G1/2)			p
MR+CT	Location	head: 38.5 %	body: 23.1 %	tail: 38.5 %	head: 29.6 %	body: 25.2 %	tail: 45.2 %	0.58
MR+CT	Median size (range)	4.2 cm (1.0 cm to 14.0 cm)			2.7 cm (0.7 cm to 15.7 cm)			**<0.01**
MR+CT	Shape	round/ ovoid: 76.9%	lobulated/ irreg.: 23.1 %		round/ ovoid: 46.1 %	lobulated/ irreg.: 53.9 %		**<0.01**
MR+CT	Volume of cystic component	≤25%: 38.4%	25-75%: 35.9%	>75%: 25.6%	≤25%: 72.2 %	25-75%: 13.9 %	>75%: 13.9 %	**<0.01**

Margin		SPN		pNEN (G1/2)		p
		**distinct**	**indistinct**	**distinct**	**indistinct**	
MR	Margin	92.3 %	7.7 %	86.9 %	13.1 %	0.72
CT	Margin	86.7 %	13.3 %	75.0 %	25.0 %	0.33

Capsule		SPN			pNEN (G1/2)			p
		**none**	**<3mm**	**≥3mm**	**none**	**<3mm**	**≥3mm**	
MR	Capsule	30.8 %	34.6 %	34.6 %	88.5 %	9.8 %	1.6 %	**<0.01**
CT	Capsule	40.0 %	53.3 %	6.7 %	92.5%	7.5 %	0.0 %	**<0.01**

Relative signal intensity/density		SPN			pNEN (G1/2)			p
		**hypo**	**iso**	**hyper**	**hypo**	**iso**	**hyper**	
MR	T1	95.5 %	0.0 %	4.5 %	94.7 %	3.5 %	1.8 %	0.74
MR	T1fs	100 %	0.0 %	0.0 %	94.0 %	4.0 %	2.0 %	1.0
MR	T2	0.0 %	4.0 %	96.0 %	1.7 %	41.7 %	56.7 %	**<0.01**
MR	T2fs	0.0 %	0.0 %	100 %	2.4 %	17.1 %	80.5 %	0.21
CT	Unenhanced	57.1 %	28.6 %	14.3 %	9.3 %	90.7 %	0.0 %	**<0.01**
MR	Arterial phase	100 %	0.0 %	0.0 %	22.6 %	26.4 %	50.9 %	**<0.01**
CT	Arterial phase	91.6 %	0.0 %	8.3 %	12.8 %	16.7 %	70.5 %	**<0.01**
MR	Portal venous phase	31.8 %	59.1 %	9.1 %	16.7 %	36.7 %	46.7 %	**<0.01**
CT	Portal venous phase	40.0 %	46.7 %	13.3 %	6.3 %	34.2 %	59.5 %	**<0.01**
MR	Delayed phase	30.0 %	50.0 %	20.0 %	16.7 %	50.0 %	33.3 %	0.34
CT	Delayed phase	0.0 %	100 %	0.0 %	0.0 %	54.5 %	45.5 %	0.26
MR	DWI (b ≥ 500 s/mm^2)	0.0 %	0.0 %	100 %	0.0 %	8.7 %	91.3 %	0.99

Homo-/heterogeneity		SPN		pNEN (G1/2)		p
		**homo**	**hetero**	**homo**	**hetero**	
MR	T1	59.1 %	40.9 %	73.7 %	26.3 %	0.21
MR	T1fs	27.8 %	72.2 %	66.0 %	34.0 %	**0.01**
MR	T2	16.0 %	84.0 %	39.0 %	61.0 %	**0.04**
MR	T2fs	14.3 %	85.7 %	39.0 %	61.0 %	0.11

Other imaging features		SPN		pNEN (G1/2)		p
		**present**	**absent**	**present**	**absent**	
MR+CT	Fluid-fluid-levels	2.6 %	97.4 %	0.0 %	100 %	0.25
MR	T1-hyperintense spots	16.0 %	84.0 %	5.0 %	95.0 %	0.19
CT	Calcification	cent.: 20.0 %<br>peri.: 6.7 %<br>both: 0.0 %	73.3 %	cent.: 7.5 %<br>peri.: 1.3 %<br>both: 6.3 %	85.0 %	0.14

Imaging features are stated for MR imaging and CT imaging. Imaging features without discrepancies between CT and MR imaging were pooled (MR+CT). Please note that the same lesion imaging features were associated with $p < 0.05$ in the analysis excluding G3 NET/NEC (this Table) and the analysis including G3 NET/NEC (Table 1 in the main document). Presented p-values are from univariate analysis. Abbreviations specific to this table: cent.: central, hetero: heterogenous, homo: homogeneous, hyper: hyperintense/-dense, hypo: hypointense/-dense, irreg.: irregular, iso: isointense/-dense, peri.: peripheral.

Supplementary Table 5. Associated imaging features of SPN and pNEN (excluding G3 NET/NEC)

Modality	Feature	SPN		pNEN (G1/2)		p
		present	absent	present	absent	
MR+CT	Vessel encasement	5.1 %	94.9 %	19.1 %	80.9 %	**0.04**
MR+CT	Vessel occlusion	FT: 0.0 %<br>TT: 0.0 %<br>w/oT: 7.7 %	92.3 %	FT: 0.0 %<br>TT: 4.3 %<br>w/oT: 12.2 %	83.5 %	0.29
MR+CT	Organ invasion	0.0 %	100 %	2.6 %	97.4 %	0.57
MR+CT	Upstream dilatation of common bile duct	0.0 %	100 %	18.9 %	81.1 %	0.08
MR+CT	Upstream dilatation of main pancreatic duct	3.6 %	96.4 %	24.2 %	75.8 %	**0.02**
MR+CT	Atrophy of upstream parenchyma	0.0 %	100 %	26.4 %	73.6 %	**<0.01**
MR+CT	Multiple lesions	0.0 %	100 %	8.7 %	91.3 %	0.07
MR+CT	Lymph node enlargement	0.0 %	100 %	15.7 %	84.3 %	**<0.01**
MR+CT	Liver metastases	0.0 %	100 %	single: 1.7 %<br>multiple: 9.6 %	88.7 %	0.13

All associated imaging features were not discrepant between MR and CT imaging. Therefore, associated imaging features were pooled for MR and CT imaging. Please note that the associated imaging features "Upstream dilatation of the common bile duct" and "Liver metastases" were not associated with $p \geq 0.05$ in the analysis excluding G3 NET/NEC (this Table), but with $p < 0.05$ in the analysis including G3 NET/NEC tumors (Table 2 in the main document). Presented p-values are from univariate analysis. Abbreviations specific to this table: FT: fresh thrombus, TT: tumor thrombus, w/oT: without thrombus.

Supplementary Figure 2. ROC curve for the predicted probabilities from the multivariate logistic regression model (including G3 NET/NEC)

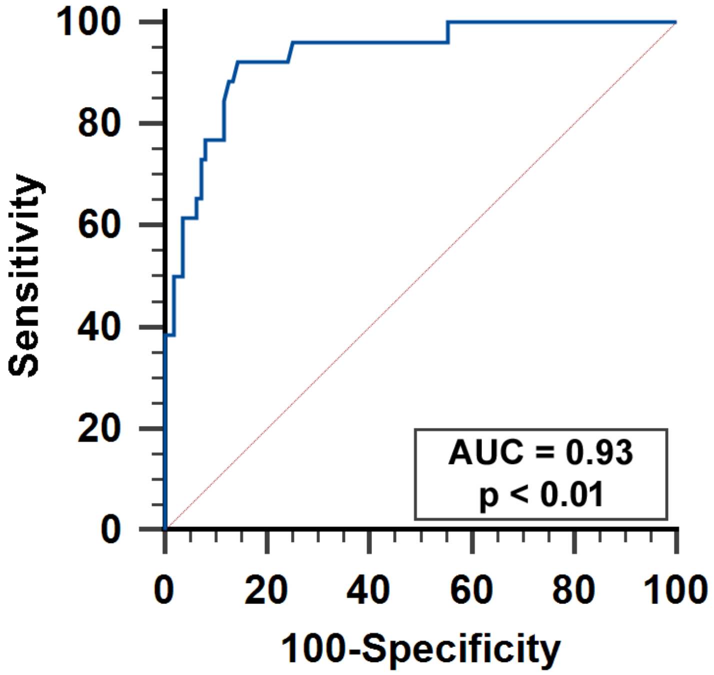

Shown is the ROC curve for the predicted probabilities from the multivariate logistic regression model for discriminating SPNs from PNENs (compare Table 3 in the main document). The predicted probability value from the model of > 0.1617 was associated with the highest Youden-index of 0.78 (95% CI: 0.63 - 0.86), Sensitivity 92.3 % (95% CI: 74.9 - 99.1%), Specificity 85.7% (95% CI: 77.8 - 91.6).

Supplementary Table 6. Multivariate analysis using logistic regression for features discriminating SPNs from pNENs (excluding G3 NET/NEC)

	Coefficient	Standard Error	P	Odds ratio (95% CI)
**Age [years]**	-0.0754	0.0249	< 0.01	0.93 (0.88 to 0.97)
**Absence of arterial hyperenhancement**	3.057	1.076	< 0.01	21.26 (2.58 to 175.14)
**Presence of a capsule**	2.289	0.690	< 0.01	9.87 (2.55 to 38.13)
**Constant**	-1.108	1.424	0.44	

Overall model significance level p < 0.01. Proportion of cases correctly classified: 86.72 %. ROC curve analysis: AUC = 0.93 (95% CI 0.88 – 0.97). Please note that the AUC from the multivariate analysis excluding G3 NET/NEC (this Table) is the same as the AUC from the multivariate analysis including G3 NET/NEC (Table 3 in the main document, Supplementary Figure 3). Variables not included in the model: sex, tumor size, tumor shape, tumor margin, volume of cystic component, venous enhancement, encasement of vessels.

Additional Reference:

[44] M.J. Eisenberg, Accuracy and predictive values in clinical decision-making, Cleve Clin J Med. 62 (1995) 311–316. https://doi.org/10.3949/ccjm.62.5.311.
